# Point‐of‐Care Ultrasound (POCUS) for Precision Management in Ellanse‐Treated Patients

**DOI:** 10.1111/jocd.70262

**Published:** 2025-05-31

**Authors:** Larry Wu, Giovanni Salti, Sebastian Cotofana, Franco Vercesi

**Affiliations:** ^1^ iCare Medical Centre Singapore Singapore; ^2^ Istituto Medico Medlight Florence Italy; ^3^ Department of Plastic Surgery Vanderbilt University Medical Center Nashville Tennessee USA; ^4^ Centre for Cutaneous Research, Blizard Institute Queen Mary University of London London UK; ^5^ Department of Plastic and Reconstructive Surgery Guangdong Second Provincial General Hospital Guangzhou China; ^6^ Centro Medico Galeno Milano Italy; ^7^ ICAMP Milano Italy; ^8^ AMEI Milano Italy

**Keywords:** biostimulator, Ellanse, facial overfill syndrome (FOS), hyaluronidase, point‐of‐care ultrasound (POCUS), transverse facial septum

## Abstract

**Background:**

Innovative use of a point‐of‐care ultrasound (POCUS) device facilitates the diagnosis and management of potential challenges associated with Ellanse treatment. Our case series of 6 patients illustrates the effectiveness of POCUS in managing challenges associated with Ellanse treatment. Ultrasound analysis is performed in all 6 patients, and management decisions are guided by ultrasound findings.

**Method:**

Our case series of 6 patients (A, B, C, D, E & F) consists of Ellanse‐treated patients. They presented problems associated with (1) facial overfilling, (2) diagnostic challenges, (3) underfilling requiring POCUS‐guided treatment, (4) confirmation of nodule dissolution, and (5) pretreatment vascular assessment.

**Results:**

Patients A and B presented with complaints of facial overfilling, and ultrasound analysis attributed the overfilling to previous autologous fat grafting and hyaluronic acid filler treatments respectively. Patient C complained of facial cheekbone prominence after receiving 26 mL of Ellanse in her entire face over 3 years. The patient was reassured that her cheek prominence was secondary to neocollagenesis following ultrasound analysis and refused further intervention. Patient D had successful dissolution of a nodule associated with Ellanse, and this was confirmed with photographs and POCUS imaging. Patient E had underdevelopment of collagen in the left midcheek following initial Ellanse treatment 3 months ago. 0.2 mL of additional Ellanse was injected with a 25G cannula under POCUS guidance to the left cheek with satisfactory correction. Patient F had three previous sessions of Ellanse treatments. She underwent vascular anatomy analysis with an ultrasound Doppler mode prior to the fourth Ellanse treatment. The presence of dense neocollagenesis following Ellanse can alter vascular patterns. A preprocedural analysis is recommended in planning subsequent treatment.

**Conclusion:**

Our limited case series of 6 patients provides useful insights into the effectiveness of POCUS as the initial imaging modality of choice when managing Ellanse‐treated patients. POCUS imaging provides valuable diagnostic information (Patient A, B, C and D) and therapeutic purposes such as POCUS guided injection and pretreatment assessment (Patient D and E). POCUS can be the initial imaging modality of choice as it is convenient and allows clinical management to be made based on imaging findings.

## 1 Introduction

Ellanse (PCL‐ELL) is a versatile collagen stimulator that is composed of 30% polycaprolactone microspheres embedded in 70% carboxymethylcellulose gel. Its rejuvenation result is dependent on fibroblast‐mediated collagen production via polycaprolactone microsphere stimulation. As PCL‐ELL is a collagen stimulator, it requires a detailed understanding of its rheology, characteristics of the filler, as well as an understanding of the injection anatomy. In addition, the lack of a hyaluronidase equivalent to dissolve inadvertently placed PCL‐ELL adds to the anxiety of the practitioner. The paradoxical formation of nodules (incidence at 0.023%) [1] which so far has a low success rate (9% success) of management further hampers the popular adoption of PCL‐ELL [2].

Point‐of‐care ultrasound (POCUS) imaging has evolved to become an essential modality in assisting practitioners address challenges associated with PCL‐ELL treated patients. Under ultrasound imaging, PCL‐ELL has the classical appearance of a hypoechoic lesion with comet‐like tails [3]. Ultrasound has proven to be tremendously useful for diagnostic and therapeutic purposes:

1. Determining if the appearance of facial overfilling is due to PCL‐ELL or other fillers.
2. Reassuring patients of the results of collagen stimulation.
3. Assessment for successful dissolution of nodule secondary to PCL‐ELL.
4. Assist in the management of inadvertent intravascular injection of PCL‐ELL [4].
5. Assist in treatment such as POCUS guided injection of PCL‐ELL and pretreatment vascular mapping.

Our case series of 6 patients illustrates the effectiveness of a POCUS device such as Clarius L20 ultra‐high‐frequency wireless ultrasound scanner (Clarius Mobile Health) in managing patients who were previously treated with PCL‐ELL.

## 2 Case 1

Patient A is a 28‐year‐old female who had five syringes (1 mL/syringe) of PCL‐ELL injected over the forehead and midcheek region. The PCL‐ELL injected is the M series, which has a product longevity of 2 years. She returned 4 years later complaining of facial overfilling. Her relevant past medical includes previous autologous fat grafting and recent pregnancy. She has experienced difficulty losing her post pregnancy fat gain on her face. In addition, she had discovered through a popular Chinese social media app “Xiao Hong Shu” (Little Red Book) that PCL‐ELL cannot be “dissolved”. As such, she was adamant that the facial fullness was attributable to PCL‐ELL treatment. Her particular concern was the prominent nasolabial fatpads. Ultrasound of the nasolabial fat pad demonstrates diffuse hypoechoeic image consistent with the appearance of fat, which does not correspond to the classical description of PCL‐ELL on ultrasound—“hypoechoeic lesion with comet like tails”. Careful documentation of treatment area, type of PCL‐ELL injected and the absence of corresponding ultrasound image confirms that PCL‐ELL is not the cause of facial overfilling.

## 3 Case 2

Patient B is a 36‐year‐old female who presented with tear trough and malar edema 1 year following treatment with Ellanse in the deep medial cheek fat (DMCF) and lateral Sub‐Orbicularis Oculi Fat (SOOF) pad. She describes the edema as puffiness, which manifests in the morning on waking up, after consuming alcohol, and after consuming a high‐sodium diet. Her relevant past medical history includes recent weight gain of 5 kg over 1 year and previous hyaluronic acid filler treatment for tear trough correction. Malar edema is described as a complication of under‐eye filler treatment and is postulated to be secondary to deposition of filler superficial to the malar septum or disruption of lymphatic drainage [5]. The hygroscopic nature of hyaluronic acid filler often contributes to delayed swelling due to water reabsorption. In certain patients who were previously treated with hyaluronic acid fillers, the subsequent injection of PCL‐ELL collagen stimulator may result in PCL‐ELL being implicated in the etiology of overfilling. An ultrasound analysis demonstrates a matrix of hypoechoic image over the SOOF with an overlying hyperechoic shadow that is consistent with previous hyaluronic acid filler treatment. She was treated with hyaluronidase with satisfactory results.

## 4 Case 3

Patient C was a 32‐year‐old female who had 26 syringes of Ellanse M injected over 3 years and she complains of malar prominence whenever she smiles. She was concerned if there was excessive collagen stimulation. Her treatment history included two milliliters of Ellanse injected in the midcheek per side to address her nasojugal and midcheek groove, which resulted in the appearance of overfilling. POCUS imaging reviews a hypoechoic image consistent with previous PCL‐ELL treatment in the DMCF compartment. After detailed counselling about addressing the collagen with triamcinolone injection and the consequent deepening of nasolabial folds, the patient decided against further intervention. She was satisfied with the reassurance of an ultrasound analysis that there is no foreign material other than PCL‐ELL induced collagen in her cheeks.

## 5 Case 4

**FIGURE 1:**
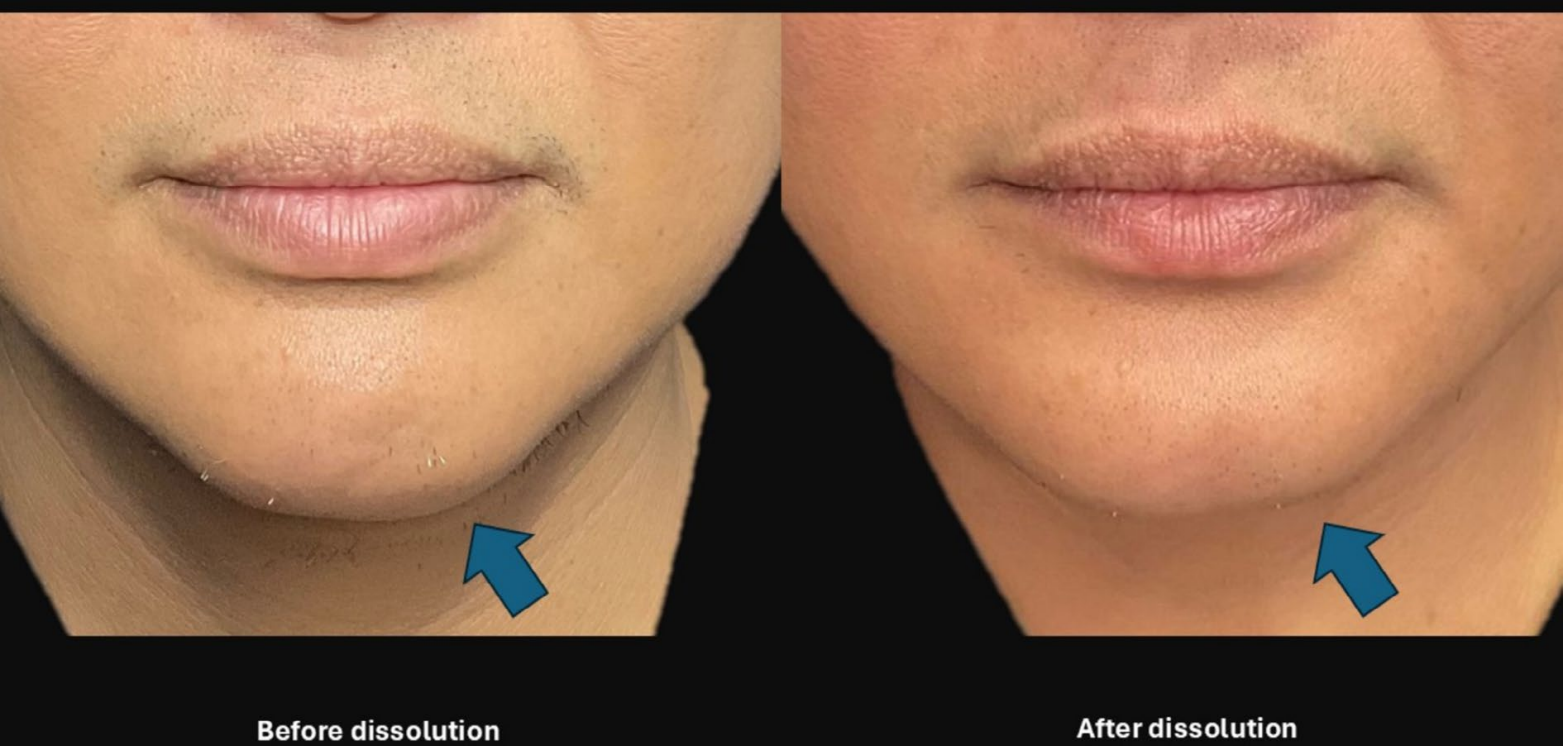
The before and after picture of chin Ellanse nodule dissolution. This can be demonstrated with picture as well as Point‐Of‐Care Ultrasound (POCUS) imaging.

Patient D was a 48‐year‐old male who developed a left chin nodule following previous treatment with PCL‐ELL. The nodule was addressed with a collagenase mixture with successful dissolution and this was confirmed with a photographic image as well as POCUS imaging (see Figure 1).

## 6 Case 5

**FIGURE 2:**
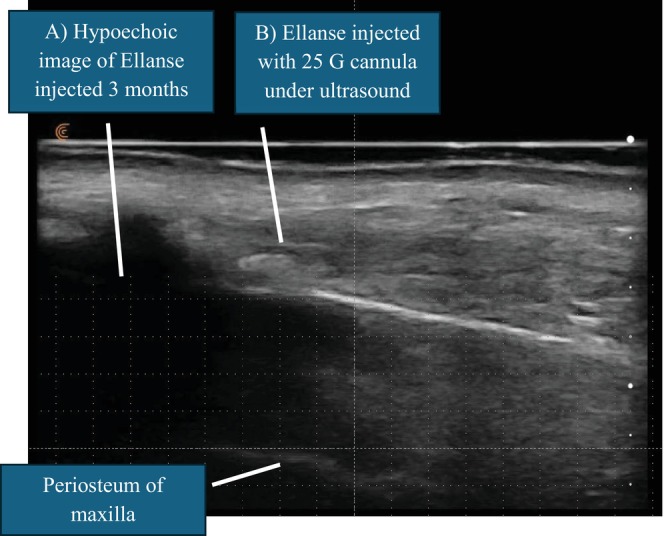
POCUS guided ultrasound‐demonstrating injection of Ellanse in previously treated area. (A) Hypoechoic image indicating previously injected Ellanse 3 months ago. (B) Ellanse injected under ultrasound guidance using a 25 G cannula (Dermasculpt).

Patient E was a 56‐year‐old female who complained of persistent left sided mid cheek hollowing following previous Ellanse treatment 3 months ago. She was treated with 0.5 mL of Ellanse in the DMCF previously, but she was dissatisfied with the inadequate collagen formation. Retreatment with Ellanse following optimal collagenesis could be challenging due to the difficulty encountered when penetrating the dense collagen with a blunt cannula. Following assessment with POCUS imaging, precise retreatment of Ellanse was performed under ultrasound guidance with 0.2 mL added adjacent to previously treated Ellanse, with satisfactory results 3 months later (see Figure 2).

## 7 Case 6

Patient F was a 49‐year‐old female who had previously undergone 3 sessions of PCL‐ELL treatment. A detailed vascular anatomy mapping was performed using Doppler ultrasound mode, and pretreatment ultrasound confirmed the presence of residual collagen from previous sessions. In patients, the vascular anatomy can be unpredictable [6] and following treatment with collagen stimulators, this can be further altered. A preprocedural mapping of blood vessels—the superficial temporal artery, supratrochlear artery, and supraorbital artery for upper face rejuvenation; the infraorbital artery and angular artery for midface rejuvenation; the facial artery, mental artery, and submental artery for lower face rejuvenation—was performed to assess the arterial pattern for safer injection. In addition, detection of residual collagen from previous PCL‐ELL treatment would prevent over injection and hence reduce the incidence of overfilling.

## 8 Discussion

Ultrasound examination is a non‐invasive imaging modality that allows real‐time visualization of patients' anatomy [7]. Ultrasound waves are generated by applying an alternating potential difference across a piezoelectric crystal to generate sound waves. Sound with a frequency above 20 kHz is known as ultrasound [8]. Ultrasound transducers use piezoelectric crystals to both generate and receive ultrasound waves. Piezoelectric literally means “pressure driven electricity” [9]. “Piezo‐” comes from Greek “piezin” meaning “to squeeze” and “electricity” comes from electron, which is the Greek word for “Amber” [10].

**FIGURE 3:**
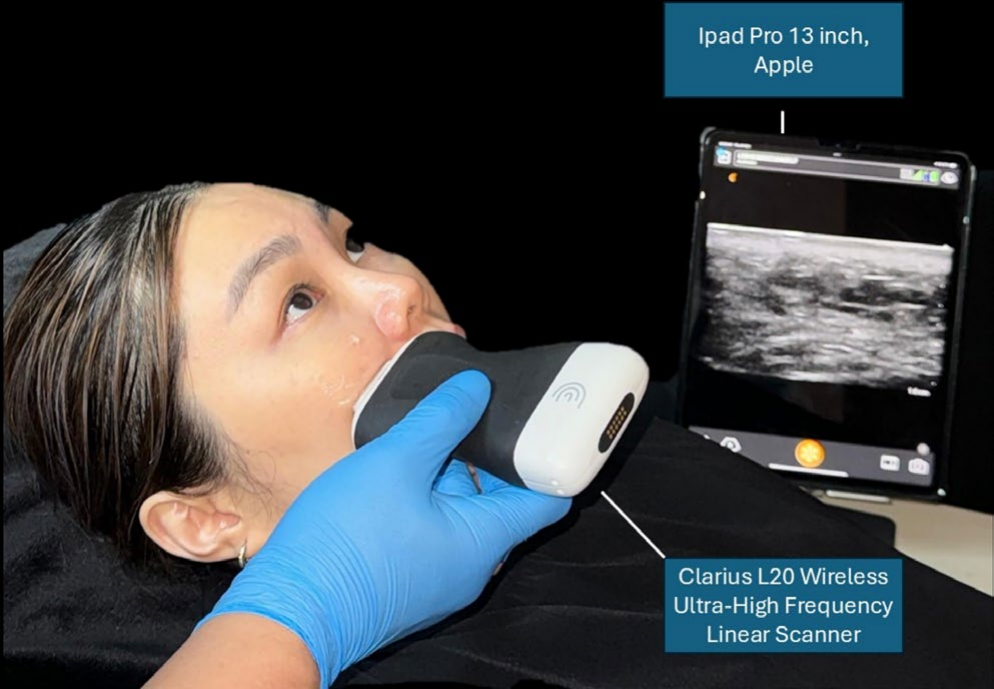
POCUS is recommended as the initial imaging modality of choice as it offers the convenience of being used in a clinic setting and the versatility of performing ultrasound‐guided treatment.

When ultrasound encounters a boundary between materials with different acoustic impedances (*Z*), a portion of the waves is reflected back (*R*) while the remainder passes through. The greater the difference in acoustic impedance between the two materials, the more waves are reflected back [11]. The reflected waves are then received by the transducer and converted back into an electric signal which can be used to generate an image. The depth of ultrasound penetration is inversely proportional to the frequency emitted by the transducer. High‐frequency transducers permit enhanced spatial resolution (greater precision) but this also results in reduced tissue penetration. For example, a portable 20 MHz device will allow high‐spatial resolution but a penetration depth up to 4 cm, which is sufficient for most facial aesthetic requirements. The use of these high‐frequency transducers enables the transcutaneous exploration of superficial tissues such as injected filler, blood vessels, and tissue layers. Ultrasound imaging has demonstrated superb capabilities in allowing real‐time analysis of injected filler material, and it allows an ultrasound‐directed treatment. Lastly, POCUS imaging is radiation‐free and is convenient as it can be performed in a clinic setting (see Figure 3).

As aesthetic procedures become more prevalent, detailed history, examination, and documentation are paramount. Patients can have previous aesthetic procedures such as hyaluronic acid fillers and autologous fat injection procedures (such as in patient A and patient B) prior to PCL‐ELL treatment. In patients who had combination treatment, the lack of a dissolution agent for PCL‐ELL coupled with the hygroscopic nature of hyaluronic acid filler [12] and fat graft hypertrophy (quoted at 4%) [13] can result in PCL‐ELL being implicated in facial overfilling [14].

As part of routine examination, if the patient has a history of previous facial filler rejuvenation, a pretreatment ultrasound is recommended to identify

1. if the stated filler corresponds to its ultrasound image,
2. the areas that were previously treated, for example, if there is accumulation of filler material over the transverse facial septum as further PCL‐ELL treatment will aggravate facial overfilling.

Meticulous documentation is also recommended and this includes

1. History of weight gain
2. Area treated with PCL‐ELL
3. Type of PCL‐ELL (S—12 months or M—24 months)

This is relevant especially when the patient reported that certain areas are overfilled and it is evidenced from the documentation that PCL‐ELL was not injected there. An ultrasound analysis would be instrumental in demonstrating the absence or presence of PCL‐ELL in the alleged area.

**FIGURE 4:**
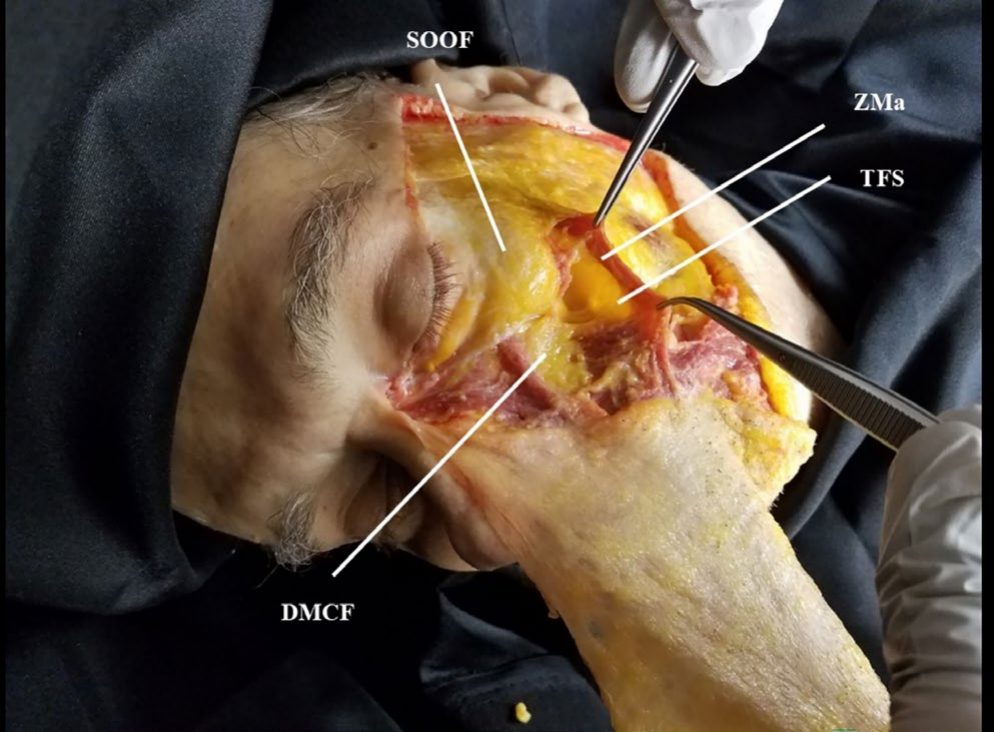
Cadaveric dissection of the left side of the face of a male body donor showing selected structures of the midface. DMCF, deep medial cheek fat compartment; TFS, transverse facial septum; ZMa, zygomaticus major muscle.

Facial Overfilled Syndrome (FOS) is a post‐treatment condition that results from excessive filler injection or improper placement, leading to an unnatural facial appearance and dynamics [15]. This often includes prominent cheeks, an unnatural smile, overfilled temples, and limited mobility around the mouth. The transverse facial septum connects to the underside of the zygomaticus major muscle (one of the primary muscles involved in smiling) and acts as the inferior boundary of both the superficial and the deep fat compartments of the midface [16]. Contraction of the zygomaticus major muscle with its underlying transverse facial septum during smiling causes the over‐injected filler located in the deep fat compartments of the midface to be cranially displaced, causing an unnatural facial appearance and facial dynamics [17]. This anatomical insight is critical for avoiding such complications and achieving a more balanced and natural result when performing facial filler treatments. The concept of the transverse facial septum and its involvement in facial biomechanics (and especially smiling) emphasizes the importance of dynamic filling and the understanding of midfacial anatomy (see Figure 4).

The presence of a bloated facial appearance has been attributed to various filler materials such as hyaluronic acid filler, poly‐L‐lactic acid, or polymethylmethacrylate (PMMA) based filler [18]. A portable ultrasound such as POCUS has the advantage of identifying the filler material that is contributing to the appearance of FOS by deciphering its characteristic ultrasonographic image in a clinic setting. These ultrasound findings facilitate clinical decision‐making, potentially reducing the need for more costly imaging modalities.

In patient E, POCUS‐guided Ellanse injection allowed a more precise placement of PCL‐ELL. This was the first known description of an ultrasound‐guided PCL‐ELL injection. In patients who had Ellanse injection within the last 6 months, the dense collagenesis can hinder penetration of the cannula and hence retreatment with PCL‐ELL can be challenging. As the patient experienced persistent underfilling in the midcheek, PCL‐ELL can be re‐injected into the region of DMCF adjacent to the dense collagenesis and hence correcting the midcheek deflation. Sterility was maintained using sterile gloves, a Betadine swab to sterilize the treatment field, and the use of a sterile imaging gel.

In patient F, who had previously 3 sessions of Ellanse treatment, POCUS allowed pretreatment mapping of vascular anatomy to facilitate safer injection. The arterial anatomy can be altered following treatment with a collagen stimulator. The dense collagen formation may encase the blood vessels, which can result in the vessels being more susceptible to injury. Pretreatment assessment allowed the vessels to be identified and to avoid inadvertent injection of PCL‐ELL.

This article has certain limitations. First, the small sample size (*n* = 6) limits the scenarios encountered by Ellanse practitioners, and a larger sample size will allow generalizability of the scenarios. The second limitation is that the images generated by POCUS necessitate correlation with a high‐resolution ultrasound performed and interpreted by qualified radiology professionals specializing in facial ultrasound. A Magnetic Resonance Imaging (MRI) could also offer a radiation‐free alternative for correlating images generated with the POCUS. Finally, histopathological analysis via biopsy of any swelling would provide independent confirmation of the ultrasound findings.

In further research regarding nodules secondary to Ellanse, the documented low incidence of Ellanse nodules (0.023% according to [1]) poses a considerable hurdle for research requiring a large patient population. Moreover, the limited availability of specialized radiographers and radiologists proficient in facial ultrasound may impact the feasibility and rigor of studies relying on advanced imaging techniques.

Future research should focus on coordinating a larger cohort of patients with complications associated with Ellanse injection. In addition, subsequent studies should correlate POCUS findings with: (1) high‐resolution ultrasound images interpreted by a qualified radiologist, and (2) histopathological analysis of excised samples.

## 9 Conclusion

The above case series illustrated challenges encountered by Ellanse practitioners. Based on our limited case series, there are certain recommendations to be made for managing patients who were previously treated with PCL‐ELL: (1) In patients with complaints of facial overfilling, POCUS is the initial imaging modality of choice to determine the causative agent. (2) For patients who require more precise injection, POCUS‐guided injection of PCL‐ELL is recommended. (3) For patients with multiple previous PCL‐ELL sessions, preprocedural ultrasound analysis is helpful in vascular mapping and confirming the presence of collagen from previous treatment. In summary, ultrasound imaging has evolved to be an essential skill that complements the existing skillset for Ellanse injectors.

## Conflicts of Interest

The authors declare no conflicts of interest.

## Data Availability

The data that support the findings of this study are available on request from the corresponding author. The data are not publicly available due to privacy or ethical restrictions.
